# Optical µ-Printing of Cellular-Scale Microscaffold Arrays for 3D Cell Culture

**DOI:** 10.1038/s41598-017-08598-3

**Published:** 2017-08-21

**Authors:** Xia Ouyang, Kunyu Zhang, Jushuai Wu, Dexter Siu-Hong Wong, Qian Feng, Liming Bian, A. Ping Zhang

**Affiliations:** 1 Photonics Research Center, Department of Electrical Engineering, The Hong Kong Polytechnic University, Hong Kong SAR, China; 2 Department of Biomedical Engineering, The Chinese University of Hong Kong, Hong Kong SAR, China

## Abstract

Guiding cell culture via engineering extracellular microenvironment has attracted tremendous attention due to its appealing potentials in the repair, maintenance, and development of tissues or even whole organs. However, conventional biofabrication technologies are usually less productive in fabricating microscale three-dimensional (3D) constructs because of the strident requirements in processing precision and complexity. Here we present an optical µ-printing technology to rapidly fabricate 3D microscaffold arrays for 3D cell culture and cell-scaffold interaction studies on a single chip. Arrays of 3D cubic microscaffolds with cubical sizes matching the single-cell size were fabricated to facilitate cell spreading on suspended microbeams so as to expose both apical and basal cell membranes. We further showed that the increasing of the cubical size of the microscaffolds led to enhanced spreading of the seeded human mesenchymal stem cells and activation of mechanosensing signaling, thereby promoting osteogenesis. Moreover, we demonstrated that the spatially selective modification of the surfaces of suspended beams with a bioactive coating (gelatin methacrylate) via an *in-situ* printing process allowed tailorable cell adhesion and spreading on the 3D microscaffolds.

## Introduction

Microscale or nanoscale topographies have revealed their essential influence on regulating cell behavior and fate^1–3^. Particularly for stem cells, their self-renewal and differentiation remarkably depend on local microenvironments, called stem cell niche^4, 5^. For instance, human mesenchymal stem cells (hMSCs) are multipotent cells and are capable of differentiating into a number of cell lineages including osteoblasts, chondrocytes, and adipocytes^6, 7^. Recent studies reported that the physical cues, including pattern size, roughness, and topography, play critical roles in stem cell fate determination of the seeded hMSCs^8–12^. However, the study of hMSCs in real three-dimensional (3D) microenvironments remains rare mainly owing to the lack of efficient approaches for massively fabricating 3D microscaffolds with tunable structural and surface properties.

The significance of cell culture in 3D extracellular matrix (ECM) mimicking realistic *in vivo* conditions was first demonstrated by Yamada *et al*.^13^. In their work, cell-derived 3D matrices were utilized to investigate cell adhesion behaviors and revealed remarkable merits of 3D migratory environments. Although these fibrous biopolymeric matrices can recapitulate some biochemical and structural features of the 3D cell microenvironments^14, 15^, these matrices do not allow precise tuning of these important cues to decouple their individual effects on cell behaviors^16^. To meet the growing demand on customization of 3D biomimetic scaffolds with composition heterogeneity^17^, a number of biofabrication technologies (e.g. inkjet bioprinting^18, 19^, microextrusion bioprinting^20, 21^, and laser-assisted bioprinting^22, 23^) have been used to fabricate 3D constructs for tissue and organ engineering^24^. For instance, the droplet-based bioprinters exhibited excellent versatility in the fabrication of 3D free-form constructs with multiple types of cells and biomaterials^25, 26^. However, the minimum feature size of droplet-based bioprinters is around 60 µm^27^, which hinders the application of these technologies in the fabrication of micrometer-scale microscaffolds for cell studies. To solve the aforementioned problem, West *et al*. proposed the use of two-photon polymerization technology to fabricate 3D biochemical and biomechanical patterns of hydrogels for guiding cell behavior^28^. Femtosecond laser pulses were focused to a small volume voxel and then scanned in three dimensions to create 3D hydrogel constructs containing micron scale features^28^. Because of its superb 3D nanofabrication capability, this laser direct-writing technology was utilized to fabricate an extensive range of 3D microscaffolds with ultrafine features for 3D cell culture studies, e.g. cell migration^29^, cell differentiation^30^, and cell morphology^31^. However, such a single-spot scanning technology is limited by its low yield in the fabrication of large volume or large quantities of ultrafine microstructures^28^. Moreover, the small two-photon absorption cross-section of photoinitiators in biomaterials requires the use of high concentrations of the photoinitiators together with high-intensity light spot to sustain polymerization efficiency^31, 32^, which therefore limits the biocompatibility of the process^33^.

An appealing solution for rapid bioprinting with micrometer-scale resolution is the use of optical maskless exposure technologies, which are capable of microengineering biomaterials via optical patterns with millions of pixels^34–37^. We demonstrated that a dynamic optical projection stereolithography (DOPsL) technology can quickly pattern various kinds of hydrogels with or without seeding of cells into user-defined 3D extracellular microenvironments for cell study^34, 35^. However, the hydrogel based direct-processing protocol still has considerable limitations on the fabrication of microscaffolds with suspended components, which have attracted increasing attention in 3D cell culture and biomechanics studies. In this work, we present an improved printing technology through the integration of DOPsL and machine vision metrology along with the development of new biomaterial processing protocols. A polymer material (i.e. SU-8 photoresist) with good mechanical property as well as excellent chemical resistance and nonirritant characteristics is used to fabricate a variety of 3D cubic microscaffolds, whose cubical sizes are tens of micrometers, to mimic the natural structure of bone lacunae^38^. An *in-situ* printing technique is then developed to selectively deposit gelatin methacrylate (GelMA) on the suspended beam as bioactive coating material to guide cell adhesion and spreading. Such a two-material based printing protocol offers a more biomimetic 3D microplatform with tunable biochemical and structural properties for controlled cell culture and migration studies, e.g. guided bone regeneration.

## Results

### Rapid fabrication of 3D microscaffold arrays

**Figure 1:**
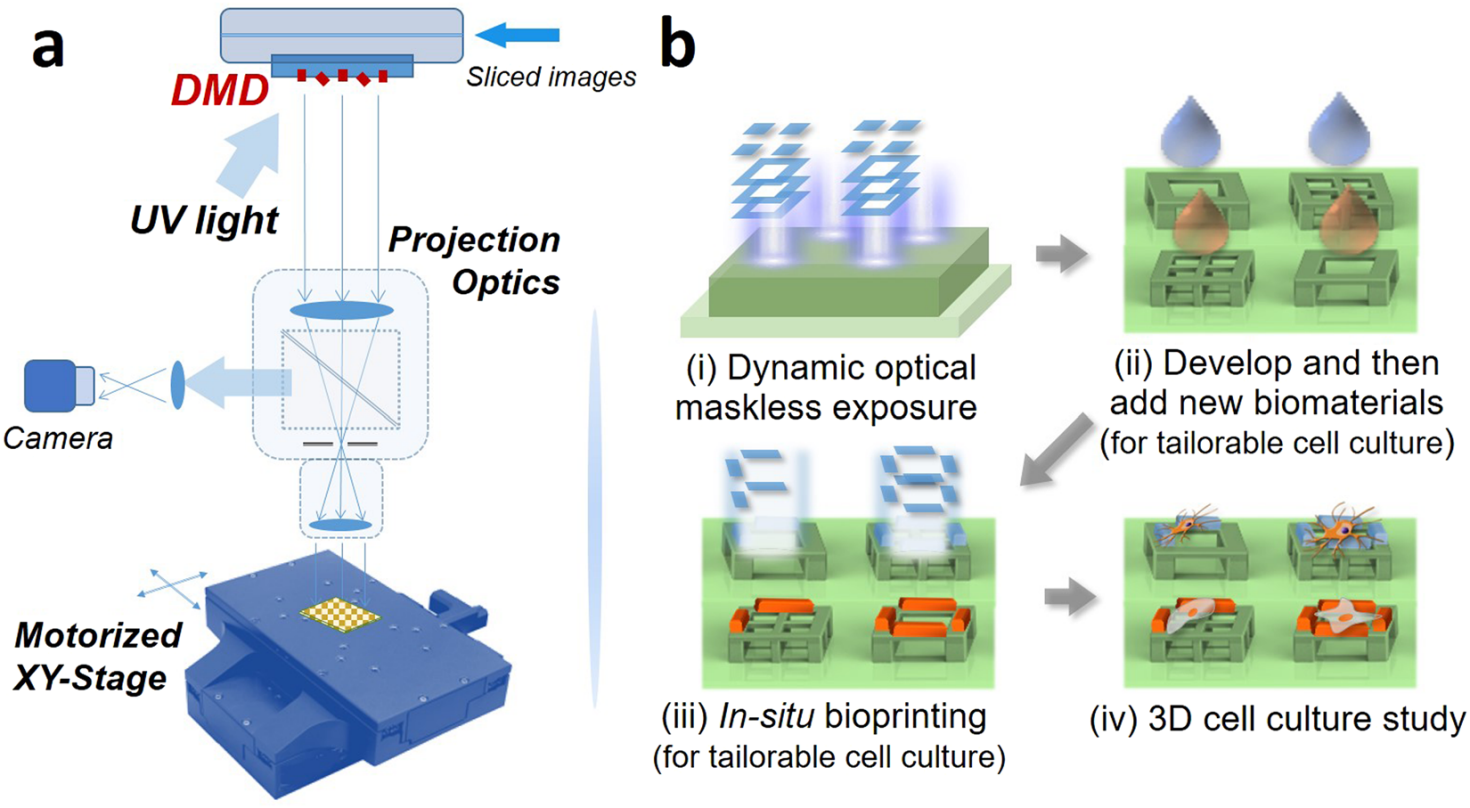
(**a**) Schematic diagram of the DMD based optical µ-printing technology. (**b**) Typical processes of the optical µ-printing technology: (i) Exposure of SU-8 photoresist via dynamic optical maskless projection technique; (ii) scaffolds developing and biomaterial coating; (iii) *in-situ* printing of biomaterials on microscaffolds; (iv) cell culture in 3D microscaffold array.

Figure 1(a) shows a schematic representation of the optical µ-printing platform based on a high-speed spatial light modulator, i.e. digital micromirror device (DMD). Compared with our previously demonstrated optical maskless exposure scheme^34, 39^, a digital camera and a XYZ-motorized stage have been further integrated to enable machine-vision metrology and high-precision alignment for *in-situ* printing processes. The 3D model of the designed scaffold is sliced into 100 layers of image data for the generation of optical patterns. The collimated UV light reflected by the DMD chip will turn into optical pattern according to the sequence of image data. As shown in Fig. 1b (i), the optical patterns will sequentially irradiate the photoresist to generate 3D microstructures based on the additive penetration property of UV light in polymer materials. The optical resolution of the system was 600 nm.

**Figure 2:**
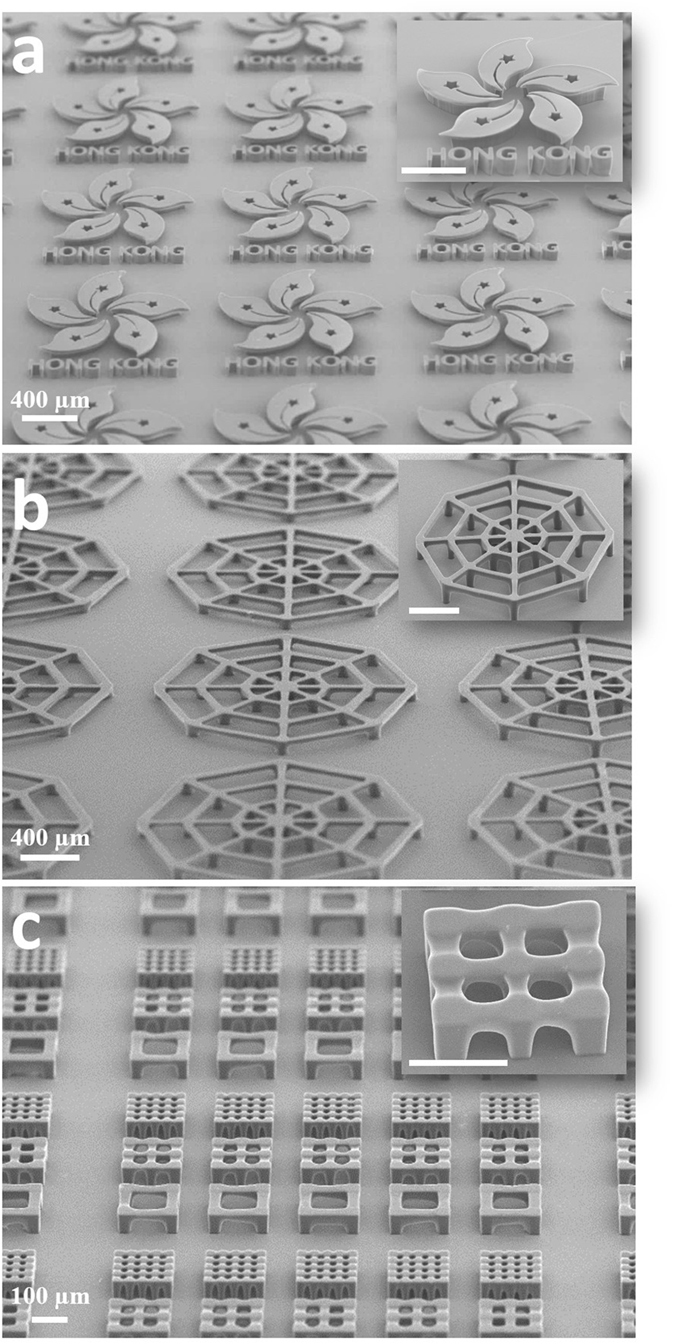
SEM images of the fabricated 3D microstructure arrays: (**a**) Hong Kong Bauhinias, (**b**) uplifted micro-cobwebs, (**c**) 3D cubic microscaffolds.

To demonstrate the versatility of the technology, we fabricated various arrays of 3D microstructure with suspended components, such as suspended Hong Kong Bauhinia (Fig. 2a), uplifted cobweb (Fig. 2b), and cubic microscaffolds (Fig. 2c), by using the photoresist SU-8. To fabricate the structure, the substrate with spin-coated SU-8 was soft baked at 65 °C for 5 min and then heated at 95 °C for 15 min to remove the solvent. Then, a UV light with an intensity of 159.12 mW/cm^2^ was applied to expose the photoresist for a total of 55 seconds, which is extremely faster than other technique like two-photon polymerization. The exposed sample was finally baked at 65 °C for 5 min, followed by a subsequent baking at 95 °C for 20 min.

It is noteworthy that these cubic microscaffolds were optimized in terms of sizes and shapes for *in vitro* cell study. As shown in Figure S1(a,b,c), the inner cubical opening size of the suspended frames of cubic scaffolds were (88 × 88), (44 × 44), and (22 × 22) µm^2^, respectively, and these cubical sizes were selected to match the size of a single cell with different extent of spreading. It can be seen that the fabricated scaffolds have the same shape with the design, except for a small bending of the suspended beams. Moreover, cubic microscaffolds of different combination modes (2 × 2 and 4 × 4) were also fabricated, as shown in Figure S1(d,e). Those microscaffolds provided a real 3D platform for e.g. the study of cell–cell interactions. The height of all microscaffolds is around 42 µm, as shown in Figure S2.

### Cell behaviors in 3D cubic microscaffolds

**Figure 3:**
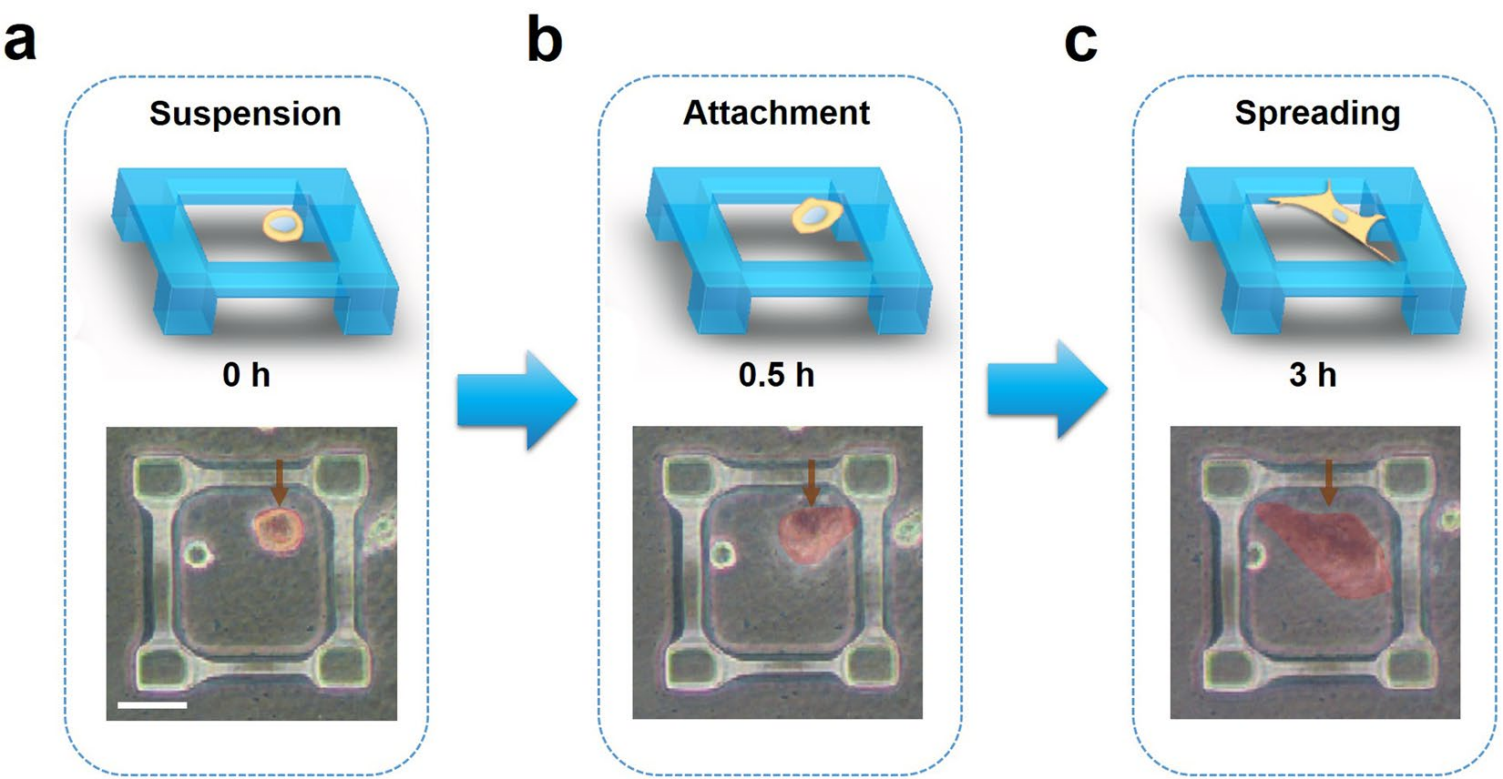
Schematic illustrations and photographed images of a single cell (highlighted in red in the bright field images) adhered to the 3D microscaffolds and its spreading over it. Scale bar = 50 μm.

After coating microscaffolds with polydopamine (PDA), hMSCs were seeded onto the 3D microscaffolds at the density of 20 000 cells/cm^2^ and continuously monitored the cells for 24 h. Right after seeding, cells were shown to get trapped in the microscaffolds (Fig. 3a). These cells then gradually attached to the beams of the cubic microscaffolds after approximately 0.5 h (Fig. 3b). It is interesting to note that the cells preferentially adhered to the suspended beams of the microscaffolds though the entire microscaffolds were pre-coated with PDA to facilitate cell adhesion. These cells subsequently elongated and spread along the beams, and an equilibrium of cell morphology reached around 3 h after the cell seeding (Fig. 3c). The control experiments showed that in the “no-beam” microscaffold array (i.e. micropillar array with the same spacing and same height as the cubic microscaffolds), cells spread only on the surface of underlying substrates but not in the space between those pillars (Figure S3). This result indicates that the suspended beams of the cubic microscaffolds are essential to facilitate cell adhesion and spreading.

**Figure 4:**
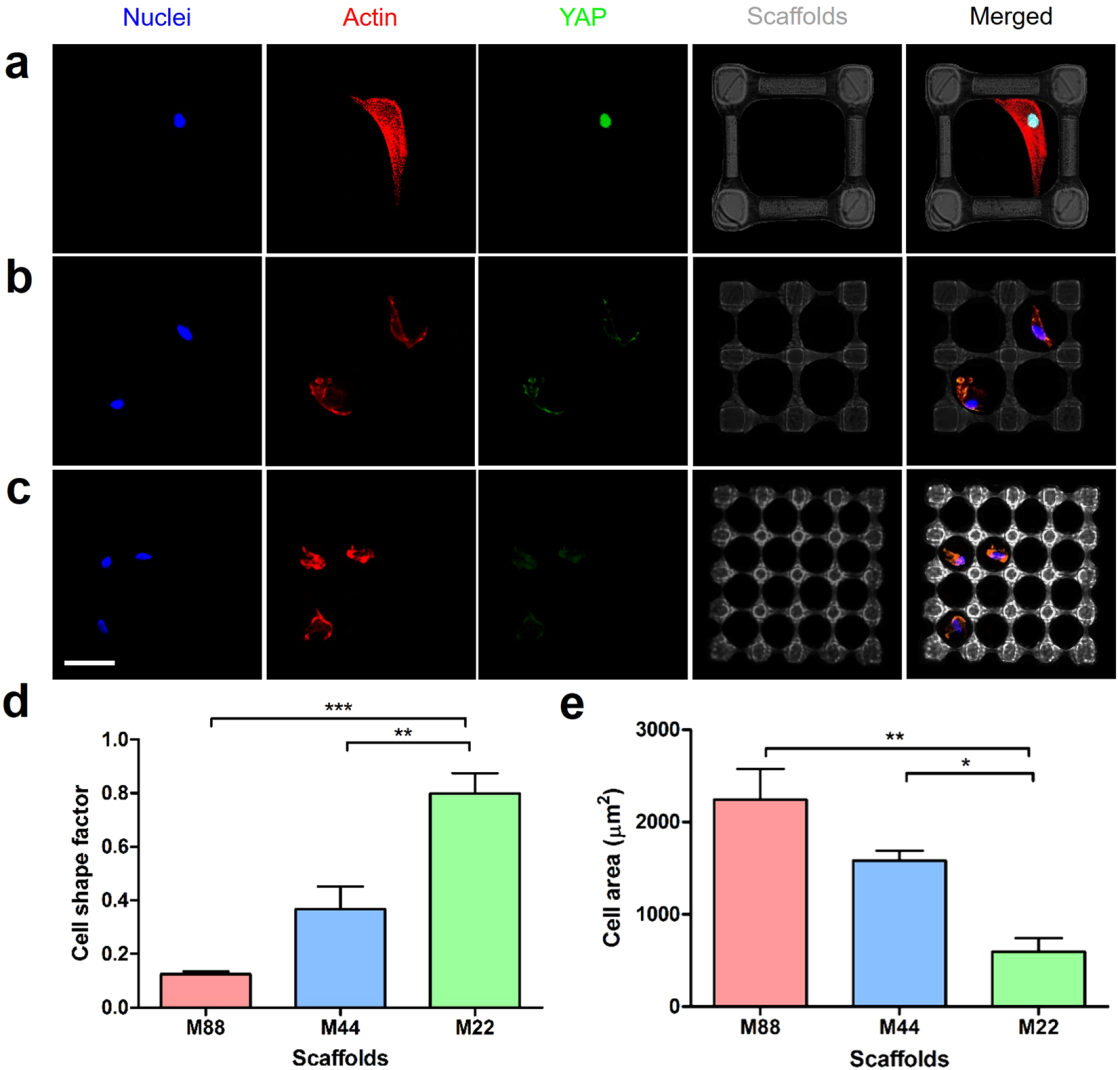
Fluorescent images of the hMSCs cultured in the 3D cubic microscaffolds with different cubicle sizes: (**a**) 88 × 88 μm^2^ (“M88”), (**b**) 44 ×44 μm^2^ (“M44”), and (**c**) 22 × 22 μm^2^ (“M22”) for 24 h; scale bar = 50 μm. (**d**) Average cell shape factors and (**e**) average cell area of hMSCs cultured in the microscaffolds. *p < 0.05, **p < 0.01, ***p < 0.001.

In order to reveal the cell morphology in such cubic microscaffolds, the actin cytoskeleton and nuclei of the hMSCs were fluorescently stained and monitored by laser scanning confocal microscopy. In the smallest single-cubicle microscaffolds with inner size of 22×22 μm^2^, the cells hardly spread and exhibited a spherical morphology. The average cell-shape-factor value (*F* = 4*πA/P* ^2^, where *A* is the area occupied by cell and *P* is the perimeter of cell) was over 0.85. With the increased size of the microscaffolds, the cells exhibited significantly more spreading, as shown in Figure S4a–d. *F*-value became close to 0.1 when the inner size of microscaffolds was 88×88 μm^2^. Furthermore, the hMSCs cultured in the larger microscaffolds showed statistically larger cell area compared with the cells in the smaller microscaffolds (Figure S4e). When seeded on the multi-cubicle microscaffolds, hMSCs generally only attached to single cubicles and spread over them and rarely spread across cubicles, and the extent of cell adhesion also showed similar trend as that observed in the single-cubicle microscaffolds (Fig. 4a–d).

Furthermore, we performed the immunostaining against yes-associated protein (YAP), which is a key mechanotransduction factor in the development of cell cytoskeletal tension. As shown in Figs 4a–c, S4a–c, the hMSCs cultured in the microscaffolds with larger cubicle sizes showed significantly brighter nuclear YAP fluorescence than the cells in the microscaffolds with smaller cubicle. After 7 d of osteogenic induction, the hMSCs cultured in the larger microscaffolds showed significantly more staining against alkaline phosphatase, a key marker of osteogenesis and biomineralization, than the cells in the smaller microscaffolds (Figure S5).

### Tailorable cell culture with *in-situ* modified 3D microscaffolds

Gelatin has been extensively used as biomaterial for tissue engineering owing to its good biocompatibility and bioactivity, such as the support on cell adhesion^40^. With the optical µ-printing technology, as shown in Fig. 1b (ii, iii, iv), the photo-crosslinkable GelMA can be selectively printed on the surface of the 3D cubic microscaffolds for guiding cell adhesion and spreading.

**Figure 5:**
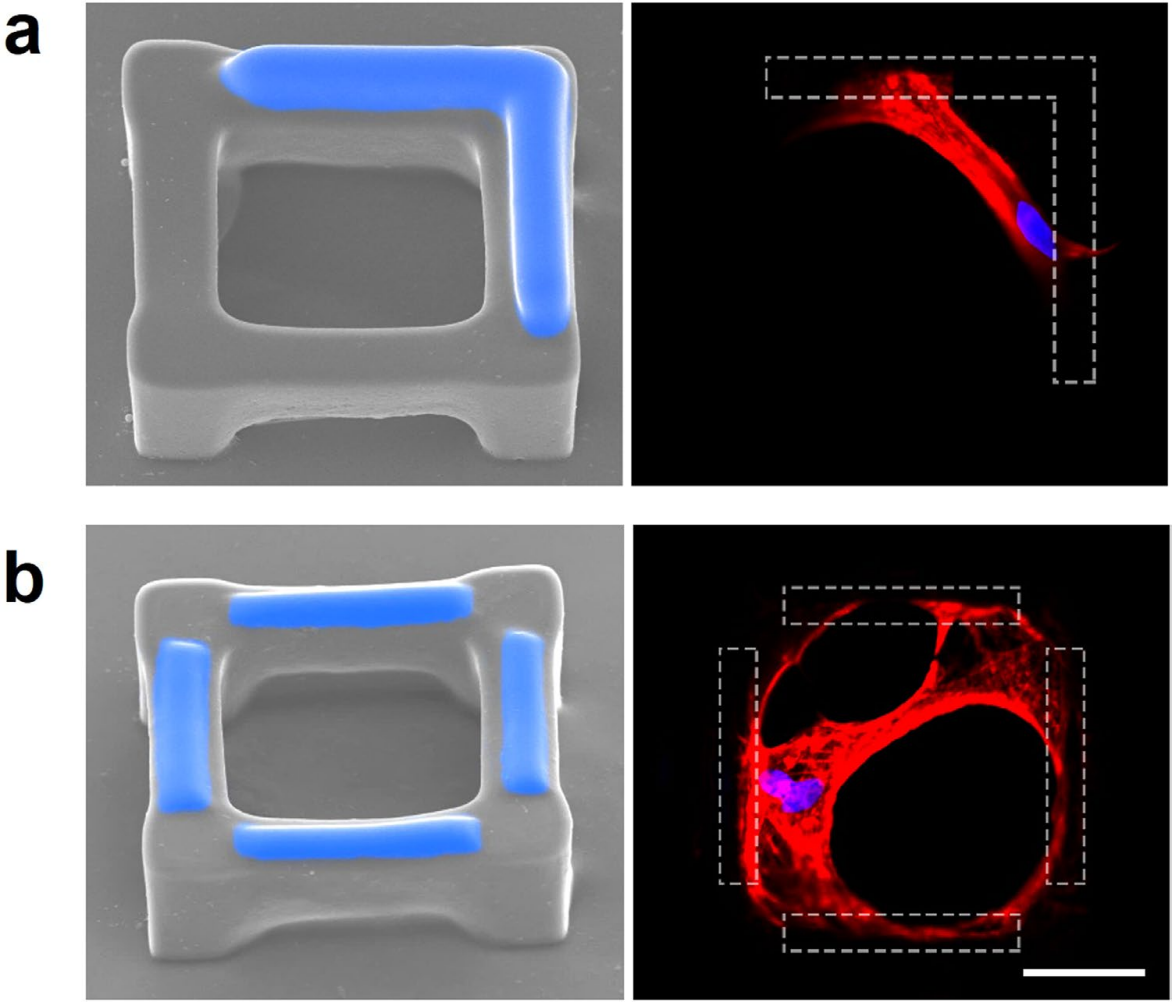
SEM images of the cubic microscaffolds with *in-situ* printed GelMA (highlighted in blue) and the fluorescent staining of f-actin (red) and nuclei (blue) of the hMSCs cultured in the corresponding microscaffolds (gelation shown as the dashed area). Scale bar = 50 μm.

Since the surface of SU-8 is hydrophobic and hard to be further coated with bioactive materials, oxygen plasma treatment was utilized to enhance the adhesion of GelMA on the SU-8 surface. The dose of plasma treatment was optimized to avoid the excessive treatment, which will recede the discrepancy of cell-attachment properties between GelMA and SU-8 surfaces^41^. The fabricated SU-8 scaffolds were treated by O_2_ plasma cleaner (PDC-32G-2, Harrick Plasma.) at low, middle, and high-radio-frequency power for 10 s separately. After plasma treatment, GelMA was dropped on the sample with the help of a pipette (Fig. 1b). Thanks to the high-precision motorized stage and the integrated machine-vision metrology, an ultrafine alignment was conducted to enable optical patterns to precisely irradiate small targets on microscaffolds. In order to polymerize GelMA only on the top surface of the suspended beams, an objective with short depth of focus (~10 μm) was used in the setup. Figure 5 and Figure S6 shows various patterns of GelMA (highlighted in blue) printed on the top of the SU-8 cubic microscaffolds. The intensity of UV light was 133.96 mW/cm^2^, and the total time of UV exposure was 30 s. The exposed samples were developed in DI water at 33 °C for 30 min.

The microscaffolds (without coating of PDA) were then used to seed hMSCs. In those 3D microscaffolds, cells barely adhered to the naked SU-8 surface, but preferentially adhered to and spread along the surface of GelMA pattern. For example, the cell spread across the corner of the cubic microscaffold when an L-shape GelMA pattern was printed on the two neighboring suspended microbeams of the scaffold (Fig. 5a). The confocal scanning of fluorescent cell staining confirmed that the cell largely adhered to the two microbeams coated with GelMA at both ends of the cell, while the central part of the cell was suspended over the internal cubicle opening of the microscaffold. When all four microbeams of the cubic microscaffold were printed with GelMA, as shown in Fig. 5b, cells were guided to adhere to all four beams and spread over the entire cubical opening of the microscaffold.

## Discussion

The control experiments revealed that the seeded hMSCs could spread across neighboring pillars with little adhesion to the surface of the supporting substrate below (Figure S3c) when the distance of two neighboring pillars was 22 μm. This finding is consistent to that previously reported by Freymann and co-workers^42^. However, when the distance was increased to 44 µm or even larger, the cells could hardly stretch across the span between the pillars and therefore mostly spread on the substrate (Figure S3a,b). The cubicle size of the microscaffolds has become the basis of choice of the microscaffolds for promoting cell spreading over suspended beams of the 3D microscaffolds instead of on the 2D substrate. The height of microscaffolds was also experimentally determined to be around 42 µm, which allowed the cells to spread on the suspended beams of the microscaffolds without adhering to the underlying substrate and thereby provided a real 3D environment for investigating cell behaviors.

In line with our observations, the further statistical analysis showed that the cell area exhibited approximately a linear correlation with the cubicle size of microscaffolds (R^2^ > 0.91, Figure S7). This phenomenon was observed in both single-cubicle and multi-cubicle microscaffolds, and this hence confirms that both types of the microscaffolds can successfully support cell spreading in 3D environments.

YAP has newly emerged as an appealing molecular sensor and mediator of mechanical cues presented by the cellular microenvironment^43, 44^. The activity of YAP is also reported to be closely involved in stem cell differentiations^45^. In particular, osteogenic differentiation of stem cells has been shown to be positively correlated with YAP activity in the cell nuclei. In this work, the hMSCs cultured in the cubic microscaffolds with larger top-opening (88 × 88 μm^2^) shows both brighter nuclear YAP fluorescence and alkaline phosphatase (ALP) activity, and this indicates the positive correlation among the size of microscaffolds, cell spreading level, YAP activity, as well as the osteogenic differentiation.

With the bioinert material, SU-8, and bioactive material, gelatin, we have demonstrated the fabrication of multi-material composite microscaffolds with spatial precision on the material deposition. SU-8 has been used to create a cell-size 3D cubic microscaffold, whereas the GelMA has been *in-situ* printed on the suspended beams of microscaffold with specific patterns. Compared with PDA coating, which lacks the precisely controlled spatial pattern, our *in-situ* printing technique can precisely control over the cell attachment site and subsequent spreading behaviors. Experimental results confirmed that cells specifically adhered to the GelMA deposited areas but not the naked SU-8 surface (Fig. 5b). Such 3D cubic microscaffolds with precisely presented bioactive cues mimic the natural structure of bone lacunae, which thus provides a very promising platform to investigate hMSC behaviors for bone research.

In summary, we have demonstrated an optical printing technology for rapid fabrication of large arrays of cellular-scale microscaffolds with suspended components for 3D cell culture and statistical cell studies on a single chip. Experiments revealed that hMSCs cultured in these 3D microscaffolds tended to adhere to the suspended beams of the microscaffolds and then spread over them. The cell morphology like shape and area profoundly depends on the geometry of the microscaffolds. Moreover, an *in-situ* optical μ-printing technology has been developed to selectively deposit GelMA on the suspended beams of microscaffold to dictate the cell adhesion to the specific structural element of the microscaffold. The strategy presented here opens a new route towards tailorable 3D cell cultures and other downstream cell studies.

## Methods

### Materials

Epon resin SU-8 was purchased from Momentive Performance Materials Inc., USA. Cyclopentanone, and 2-hydroxy-4-methoxybenzophenon-5-sulfonic acid (HMBS) and propyleneglycol monomethylether acetate were purchased from J&K Scientific, China. 2-(2H-Benzotriazol-2-yl)-4,6-bis(1-methyl-1-phenylethyl)phenol (TINUVIN 234) were ordered from Sigma–Aldrich, USA. Tributylamine (TBA) and 4-((2-hydroxytetradecyl)oxy)phenyl)-phenyliodoniu (PC-2506) were purchased from Meryer Chemical Technology, China and Polyset Company, USA, respectively. Gelatin (type A), methacrylic anhydride, 2,2,6,6-tetramethylpiperidine 1-oxyl (TEMPO, free-radical quencher), Irgacure 2959, acrylic acid (AA), bovine serum albumin (BSA), 4′, 6-diamidino-2-phenylindole (DAPI), paraformaldehyde, Triton-X 100, and phalloidin–tetramethylrhodamine B isothiocyanate (phalloidin-TRITC) were ordered from Sigma-Aldrich.

Dopamine hydrochloride was purchased from J&K Scientific. Phosphate buffered saline, α-minimum essential medium (α-MEM), penicillin, streptomycin, L-glutamine, Calcein-AM, and fetal bovine serum (FBS) were obtained from Gibco, USA. hMSCs were obtained from Lonza. The water used in all the experiments was purified by Millipore system.

### Optical µ-printing setup

The optical µ-printing platform consists of six parts: UV light source, DMD chip, projection optics, digital camera, motorized stage and computer for sliced images. The UV light source (L10561) was purchased from Hamamatsu Photonics K.K., Japan. The DMD chip and its control board (DLP9500) were purchased from Digital Light Innovations (DLi), USA. The lenses in the projection optics were purchased from Thorlabs Inc., USA. The motorized stage (ANT130-XY) was purchased from Aerotech Inc., USA. An in-house add-on software in commercial 2D/3D data analysis and visualization platform (Tecplot Inc., USA) was used to generate sliced images for optical exposure, and an own-developed system control software integrated with machine-vision module was used to enable automated optical μ-printing processes.

### Preparation of SU-8 photoresist

PC-2506 and TBA are used as the photo initiator and inhibitor, respectively. TINUVIN 234 was used as UV absorber agent to control the penetration depth of UV light. Taking cyclopentanone as solvent, SU-8 photoresist was prepared by mixing Epon resin SU-8, PC-2506, TBA, and TINUVIN 234 in a weight ratio of 100: 2.5: 0.14: 0.2.

### Surface coating with PDA

In order to improve the cell attachment and adhesion on the microscaffolds, an adhesive PDA layer was coated onto the surface of the microscaffolds. Briefly, the surface was immersed in dopamine solution (1 mg/ml in Tris–HCl buffer, pH = 8.5) and incubated for 4 h, rinsed with PBS, and sterilized by UV irradiation prior to the cell experiments.

### Preparation of GelMA

To synthesize GelMA, 10 g of gelatin (type A) was dissolved in 100 ml PBS at 50 °C. Methacrylic anhydride (12 ml) was then added and the reaction was allowed to proceed for 4 h at 50 °C with continuous stirring. The resulting mixture was dialyzed against DI water for 6 d at 45 °C and then lyophilized. The degree of substitution determined by^1^H NMR was 3.17 × 10^−4^ mol/g. Irgacure 2959, HMBS and TEMPO were used as the photo initiator. UV-absorber agents, and photo inhibitor in 13% GelMA respectively. To prepare 13% GelMA solution, 0.2 g GelMA was dissolved in 1 ml DI water at 35 °C for 6 h. Then 0.03 g AA, 0.02 g Irgacure 2959, 0.0045 g TEMPO and 0.005 g HMBS were mixed with the GelMA solution and stirred at 35 °C for 12 h.

### Cell culture

hMSCs were expanded to passage 4 in growth medium containing α-MEM with 16.7% FBS, 1% glutamine and 1% pen/strep. The cells were then seeded at a constant density of 20 000 cells/cm^2^ onto the microscaffolds and incubated at 37 °C in growth medium for 48 h. For osteogenic induction, cells were cultured in growth medium for 24 h, and then in the osteogenic medium (α-MEM, 16.67% FBS, 1% glutamine, 1% pen/strep, 10 mM β-glycerophosphate disodium, 50 mg/ml ascorbate, 0.1 μM dexamethasone) for 7 d. Medium changes were performed every 2 d.

### Fluorescence staining and image analysis

Briefly, cells were fixed with 4% paraformaldehyde solution for 20 min at room temperature, rinsed with PBS three times, permeabilized with 2% Triton-X100 for 30 min at room temperature, and then blocked with 1% BSA at 37 °C for another 30 min. For the immunofluorescent staining, the cells were stained with primary antibodies against YAP at 4 °C overnight, and the secondary antibody labeling was performed by incubation in goat anti-mouse IgG containing Alexa488 for 2 h at room temperature. Then the cytoskeleton was stained with phalloidin-TRITC, and cell nuclei were stained with DAPI. Fluorescent images were acquired with a Nikon C2+ confocal microscope, and analyzed using Image J (NIH). Over 20 cells per group were counted.

### Statistical analysis

All data are presented as mean ± standard deviation. Statistical analysis was performed by using one-way ANOVA and Tukey’s post hoc testing. Tests were conducted with a 95% confidence interval (α = 0.05).

## Electronic supplementary material

Supplementary Information
