# Supplementary material for: Optical µ-Printing of Cellular-Scale Microscaffold Arrays for 3D Cell Culture
Source: Sci Rep. 2017 Aug 21;7:8880. doi: 10.1038/s41598-017-08598-3 (PMC5566436; doi:10.1038/s41598-017-08598-3)
Supplement: Supplementary file 1 — Supplementary Information [file 41598_2017_8598_MOESM1_ESM.doc]

# Title: Optical µ-Printing of Cellular-Scale Microscaffold Arrays for 3D Cell Culture

**Authors**: Xia Ouyang^1*^, Kunyu Zhang^2*^, Jushuai Wu^1^, Dexter Siu-Hong Wong^2^, Qian Feng^2^, Liming Bian^2^, & A. Ping Zhang^1^

# SUPPLEMENTARY FIGURE LEGENDS:

**Figure S1.** The designed structures and the corresponding SEM images of the 3D cubic microscaffolds. Scale bar = 50 μm.

**Figure S2.** The laser-scanning confocal images of the fabricated SU-8 microscaffolds and micropillars. The heights of the microstructures from (a) to (f) are 41.88, 42.75, 41.58, 42.49, 40.77, and 41.66 μm, respectively.

^1^Photonics Research Center, Department of Electrical Engineering, The Hong Kong Polytechnic University, Hong Kong SAR, China. ^2^Division of Biomedical Engineering, Department of Mechanical and Automation Engineering, The Chinese University of Hong Kong, Hong Kong SAR, China. Correspondence and requests for materials should be addressed to A.P.Z. (email: [azhang@polyu.edu.hk](mailto:azhang@polyu.edu.hk)) or to L. B. ([lbian@mae.cuhk.edu.hk](mailto:lbian@mae.cuhk.edu.hk)).

*These authors contributed equally to this work.

**Figure S3.** Fluorescent images of the hMSCs cultured in 3D micropillar arrays for 24 h. The cells bypassed the pillars and randomly spread. Scale bar = 50 μm.

**Figure S4.** Fluorescent images of the hMSCs cultured in the single-cubicle 3D microscaffolds with different cubicle sizes. (a) 88×88 μm^2^ (“S88”), (b) 44×44 μm^2^ (“S44”), and (c) 22×22 μm^2^ (“S22”) for 24 h; scale bar = 50 μm. (d) Average cell shape factors and (e) average cell area of hMSCs cultured in the microscaffolds; ****p* < 0.001.

**Figure S5.** Alkaline phosphatase staining of the hMSCs cultured in (a) single-cubicle 3D microscaffolds with different cubicle sizes: 88×88 μm^2^ (“S88”), 44×44 μm^2^ (“S44”), and 22×22 μm^2^ (“S22”), and (b) multi-cubicle 3D microscaffolds with different cubicle sizes: 88×88 μm^2^ (“M88”), 44×44 μm^2^ (“M44”), and 22×22 μm^2^ (“M22”) after osteogenic induction for 7 d; scale bar = 50 μm. (c) Average ALP activity of the hMSCs; ***p* < 0.01, ****p* < 0.001.

**Figure S6.** SEM images of the 3D cubic microscaffolds with *in-situ* printed GelMA patterns (highlighted in blue). Scale bar = 50 μm.

**Figure S7.** The dependence of cell areas on the top-opening sizes of cubic microscaffolds.


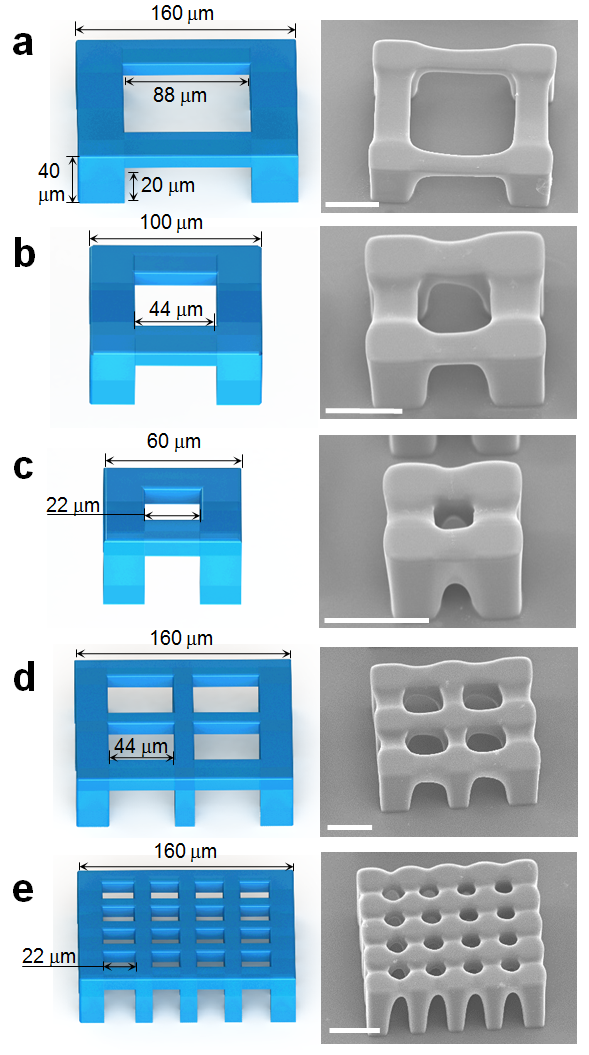


**Figure S1** (by Xia Ouyang et al.)


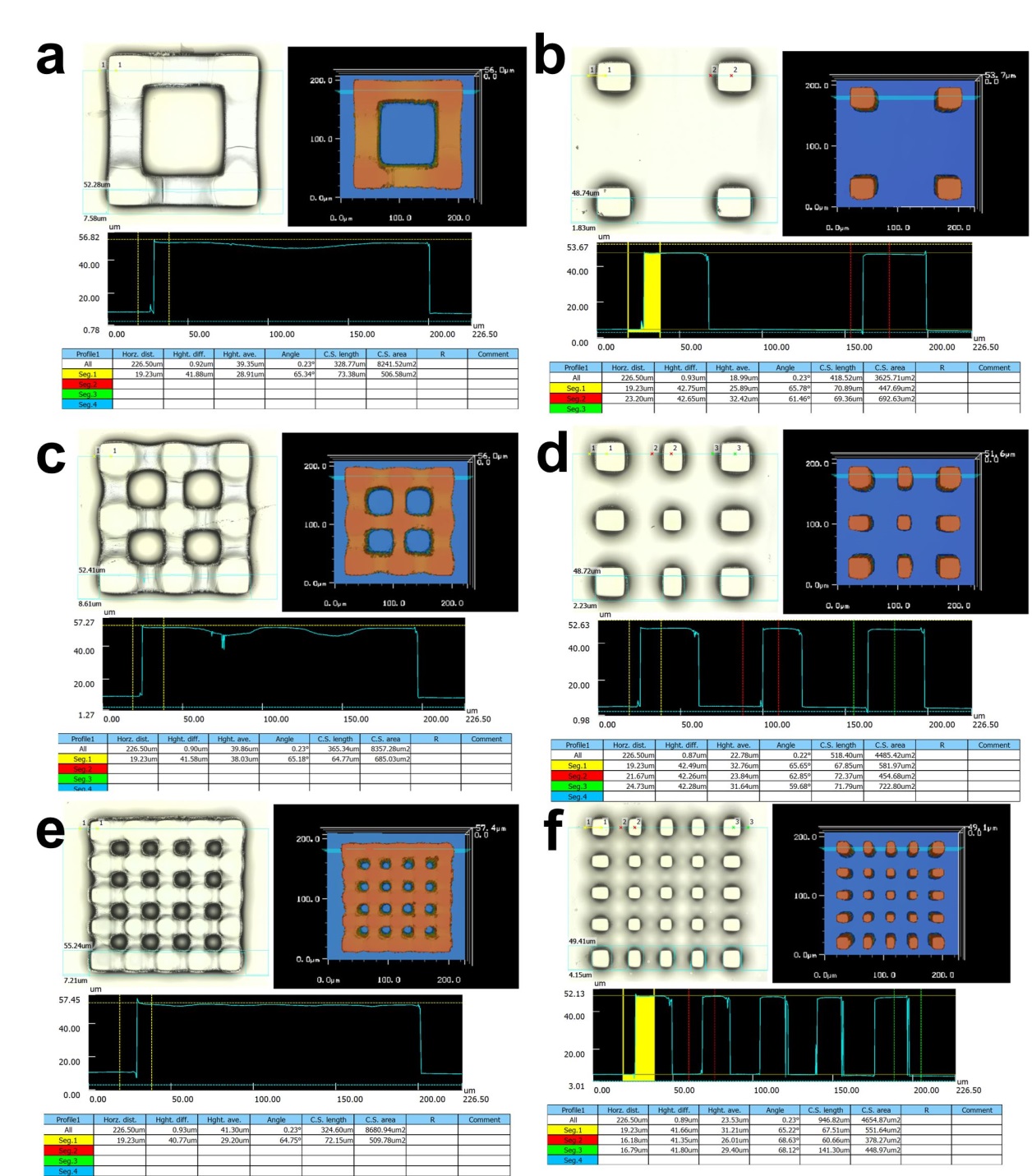


**Figure S2** (by Xia Ouyang et al.)


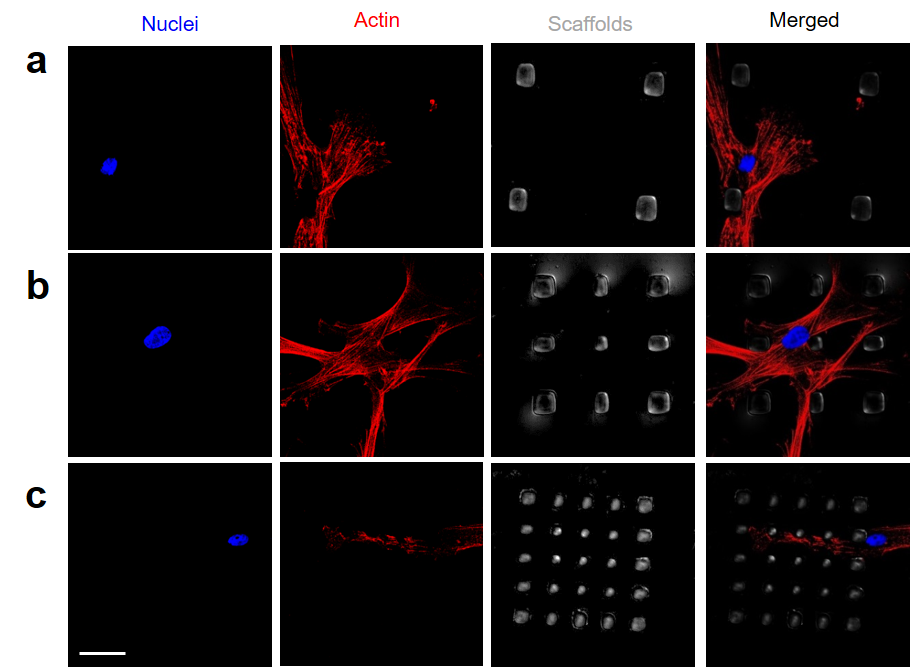

**Figure S3** (by Xia Ouyang et al.)


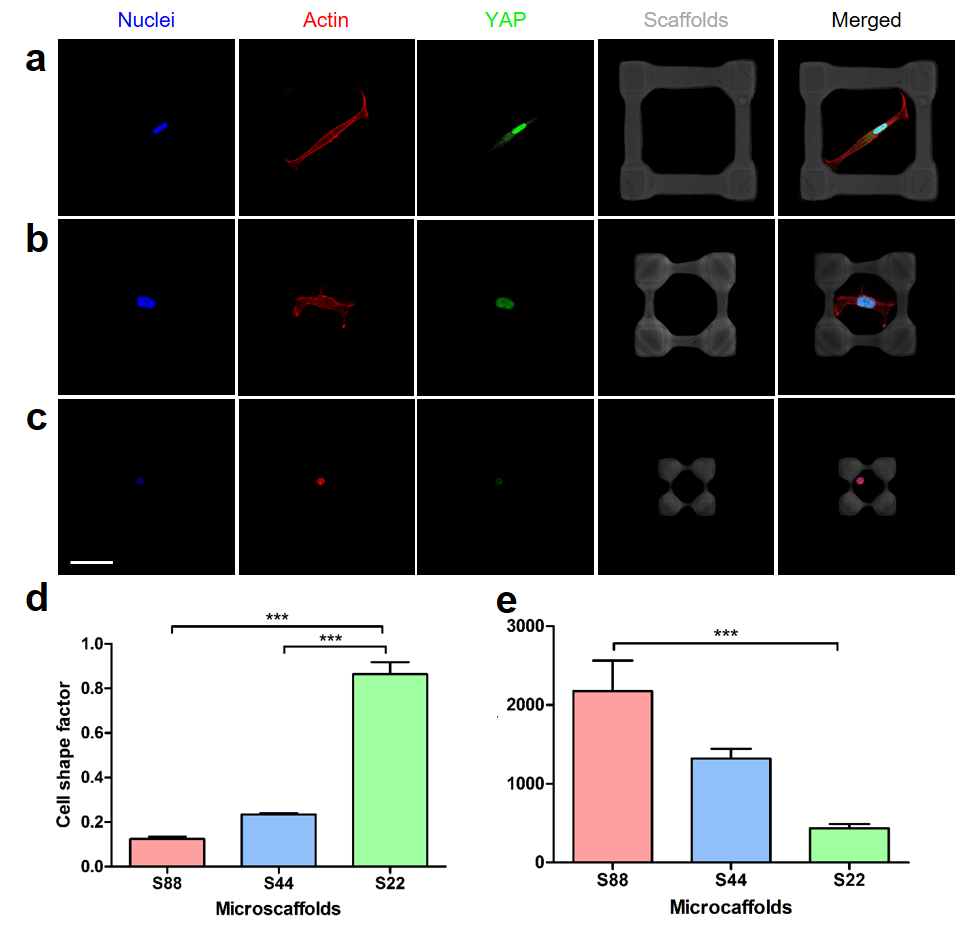


**Figure S4** (by Xia Ouyang et al.)

**
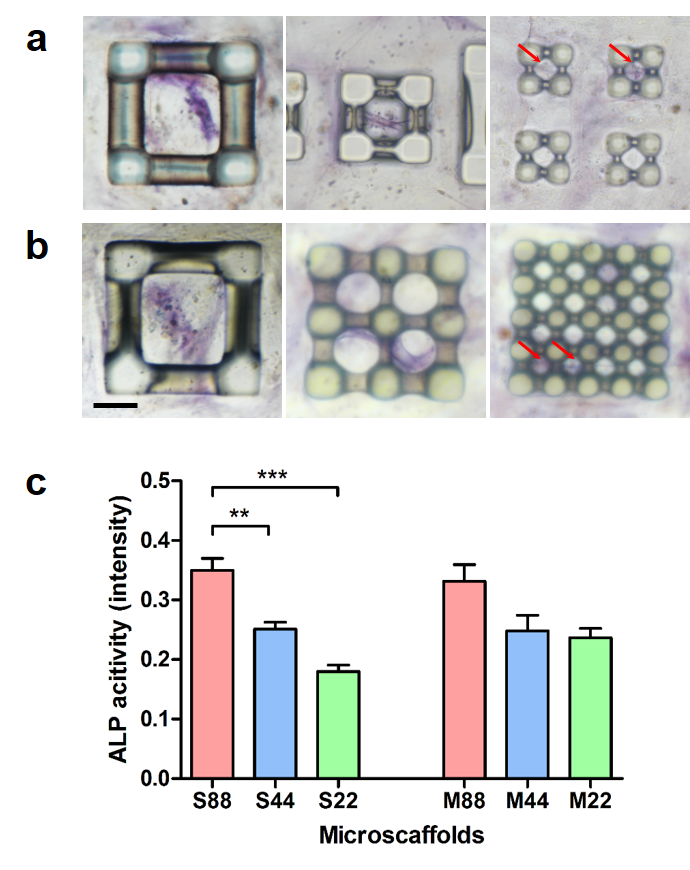
**

**Figure S5** (by Xia Ouyang et al.)


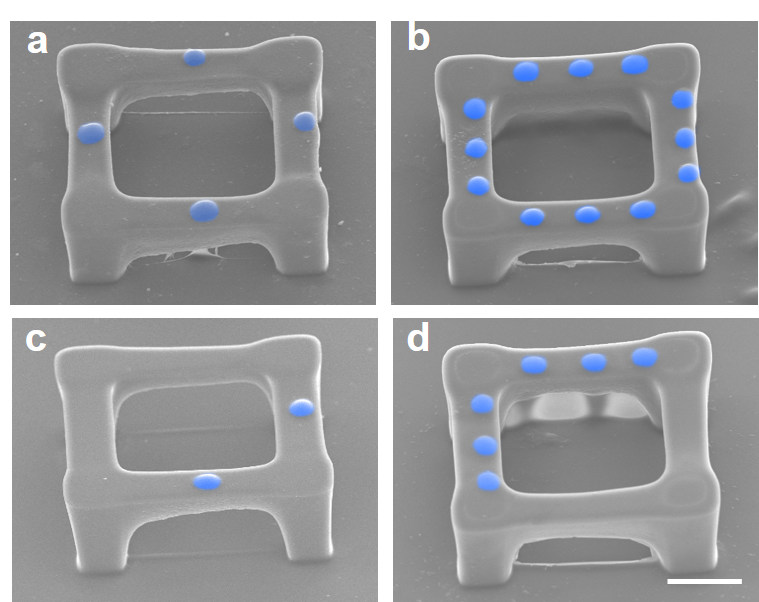


**Figure S6** (by Xia Ouyang et al.)


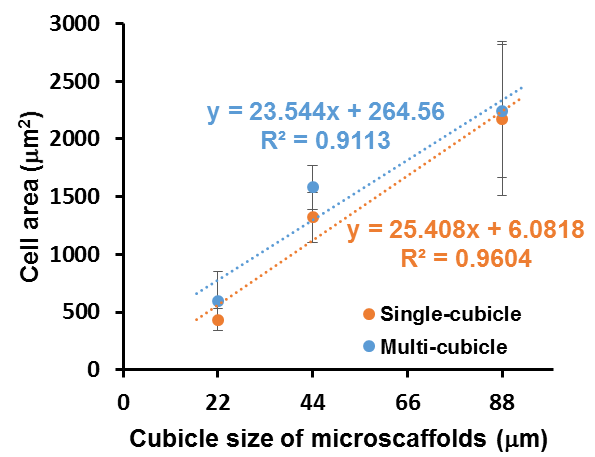


**Figure S7** (by Xia Ouyang et al.)
